# CD103 Promotes the Pro-inflammatory Response of Gastric Resident CD4^+^ T Cell in *Helicobacter pylori*-Positive Gastritis

**DOI:** 10.3389/fcimb.2020.00436

**Published:** 2020-08-21

**Authors:** Peiyu Chen, Siqi Ming, Juanfeng Lao, Chunna Li, Hongli Wang, Liya Xiong, Shunxian Zhang, Zibin Liang, Xiaoli Niu, Simei Deng, Lanlan Geng, Minhao Wu, Yongjian Wu, Sitang Gong

**Affiliations:** ^1^Department of Gastroenterology, Guangzhou Women and Children's Medical Center, Guangzhou Institute of Pediatrics, Guangzhou Medical University, Guangzhou, China; ^2^Center for Infection and Immunity, Zhongshan School of Medicine, The Fifth Affiliated Hospital, Sun Yat-sen University, Guangzhou, China

**Keywords:** CD103, *Helicobacter pylori*, gastritis, CD4^+^ T cells, TCR signal

## Abstract

CD103 is considered as a surface marker for the resident immune cells. However, little is known about the intrinsic function of CD103 in infection and inflammation. In this study, we found that CD103 was highly expressed in CD4^+^T cells of the gastric mucosa from patients with *H. pylori*-positive gastritis. Mucosal resident CD103^+^CD4^+^T cells exhibited an increase in the CD45RO^+^CCR7^−^ effector memory phenotype and high expression of the chemokine receptors CXCR3 and CCR9 compared with those in CD103^−^CD4^+^T cells. An *In vitro* coculture study demonstrated that *H. pylori*-specific antigen CagA/VacA-primed dendritic cells (DCs) induced proliferation and IFN-γ, TNF as well as IL-17 production by CD103^+^CD4^+^T cells from patients with *H. pylori*-positive gastritis, while blocking CD103 with a neutralizing antibody reduced proliferation and IFN-γ, TNF, and IL-17 production by CD103^+^CD4^+^T cells cocultured with DCs. Moreover, immunoprecipitation revealed that CD103 interacted with TCR α/β and CD3ζ, and activation of CD103 enhanced the phosphorylation of ZAP70 induced by the TCR signal. Finally, increased T-bet and Blimp1 levels were also observed in CD103^+^CD4^+^T cells, and activating CD103 increased T-bet and Blimp1 expression in CD4^+^T cells. Our results explored the intrinsic function of CD103 in gastric T cells from patients with *H. pylori*-positive gastritis, which may provide a therapeutic target for the treatment of gastritis.

## Introduction

*Helicobacter pylori* (*H. pylori*) is a well-recognized gastric pathogenic bacterium that infects more than 50% of the world's population. *H. pylori* infection is predominantly acquired in childhood, persisting throughout life, and increases susceptibility to digestive diseases, such as peptic ulcers and gastric carcinoma (Correa and Piazuelo, 2008). The clinical outcome of sustained gastrointestinal infection by *H. pylori* is determined by the reciprocal interactions between the virulence factors of the bacterium and the host immune response (Jafarzadeh et al., 2018).

CD4^+^ T cells play a considerable role in *H. pylori* infection, and their responses can promote local inflammation, can be protective and can contribute to the immune response toward the bacterium (Larussa et al., 2015). A previous study indicated that the adoptive transfer of CD4^+^ T cell to immune-deficient mice was associated with a serious and rapid development of complications (Eaton et al., 2001). In humans, the T helper (Th) effector cells-related immune responses elicited against *H. pylori* strongly tend toward Th1 polarization. Interferon-γ (IFN-γ and tumor necrosis factor (TNF) represent the main cytokines involved in the promotion of *H. pylori*-specific Th1 cells (Haeberle et al., 1997; Luzza et al., 2001; Pellicano et al., 2007). Th1 cytokines cause the recruitment of macrophages to the infection site (Jager and Kuchroo, 2010) and (especially IFN-γ) activate macrophages to fuse lysosomes more efficiently to phagosomes, which strongly contribute to the eradication of *H. pylori* (Wilson and Crabtree, 2007). Experimentally, Th1 cell-related responses have been associated with gastritis, because a reduced degree of inflammation has been documented in the stomach of IFN-γ^−/−^ mice (Smythies et al., 2000; Eaton et al., 2006). Th17 cell-related pro-inflammatory cytokines (IL-17a and IL-17f) also contribute to gastritis (Gray et al., 2013), while Th1-independent gastritis could be ascribed to the hyper-activation of the Th17 cell subset (Eaton et al., 2006). IL-17a induces the secretion of the neutrophil chemokine IL-8 from DCs and macrophages, and is involved in *H. pylori*-induced gastric mucosal inflammation (Kabir, 2011; Bagheri et al., 2015). Moreover, IL-17-stimulated gastric epithelial cells produce the matrix metalloproteinase MMP-9 and the chemokines CCL25 and CXCL8 following contact with *H. pylori*, which contribute to tissue damage of gastritis and the infiltration of inflammatory cells (such as macrophage and neutrophil) (Shi et al., 2010). The accumulation of Th17 cells in *H. pylori*-infected tissues could represent a source of chronic inflammatory injury promoting carcinogenesis (Ohata et al., 2004). Cytotoxin-associated gene A (CagA) and vacuolating cytotoxin A (VacA) proteins have been identified as virulence factors displayed by *H. pylori* and are considered the main virulence factors that cause the development of severe gastric lesions and immune responses (Yong et al., 2015; Sinnett et al., 2016). CagA-positive *H. pylori* have been found to induce more severe mucosal damages and inflammatory responses (Bassagh et al., 2019). Increased gastric VacA expression during *H. pylori* infection is associated with inflammation and premalignant pathology (Sinnett et al., 2016). IFN-γ expression in gastric biopsies varies depending on the *H. pylori* vacA and cagA genotype (Martinez-Carrillo et al., 2014). In particular, *H. pylori* infection promotes the Th17 immune response through the virulence factor CagA (Lina et al., 2013). These studies indicated that both IFN-γ, TNF, and IL-17a play important roles in *H. pylori* infection.

CD103 (the α-chain of the integrin αEβ7) is the predominant integrin expressed by mucosal resident T cells, and plays an important role in the homeostasis of T cells by modulating selective retention in the intestinal epithelia and lamina propria through binding to E-cadherin (Sheridan and Lefrancois, 2011). The numbers of mucosal resident T cells but not spleen T cells were reduced in CD103-deficient mice (Schon et al., 1999). Blockage of CD103 by anti-αEβ7 antibodies suppressed the accumulation of T cells in the gut mucosa (Zundler et al., 2017). Furthermore, a potential pro-inflammatory role (IL-17a and IFN-γ) of the CD103^+^CD4^+^ subset was reported in in ulcerative colitis (Lamb et al., 2017). Except for adhesion, recent studies have indicated that CD103 has a regulatory function for T cells (especially CD8^+^T cells) (Corgnac et al., 2018; Hardenberg et al., 2018). CD103-deficient CD8^+^ T cells were strikingly defective in transferring intestinal GVHD pathology and mortality (El-Asady et al., 2005). In kidney transplants in rats, the inhibition of CD103 attenuated tubular injury, and CD103-deficient CD8^+^T cells did not damage the tubular epithelium (Yuan et al., 2005). Anti-CD103 neutralizing monoclonal antibodies compromise the cytotoxic function of CD103^+^ tumor-infiltrating lymphocytes (TILs) to kill autologous tumor cells (Djenidi et al., 2015). However, CD103 appears to be related to the anti-inflammatory activities of CD4^+^ T_reg_ (Chang et al., 2012; Braun et al., 2015). CD103-expressing CD4^+^ T_reg_ feature higher levels of CTLA-4, ICOS, GITR, CD69, granzyme B, FasL, and CCR5, which are important for immunomodulatory activity (Lin et al., 2009; Chang et al., 2012). CD103 was crucial for the regulation of murine contact hypersensitivity, as CD103-deficient T_reg_ are unable to suppress allergic skin inflammation (Braun et al., 2015). Thus, the intrinsic role of CD103 in T cell response remained unclear, which may be needed for further study.

In this study, we explored the expression and function of CD103 in mucosal resident CD4^+^T cells from patients with *H. pylori*-positive gastritis. CD103 was associated with the differentiation, proliferation and cytokine production og gastric CD4^+^T cells. More importantly, we identified that CD103 interacted with TCRα/β and enhanced CD3ζ/ZAP70 signaling, which are essential for proliferation and pro-inflammatory cytokine production by gastric CD4^+^T cells.

## Results

### Numbers of CD103^+^CD4^+^T Cells Were Increased in the Gastric Mucosa of Patients With *H. pylori*-Positive Gastritis

**Figure 1:**
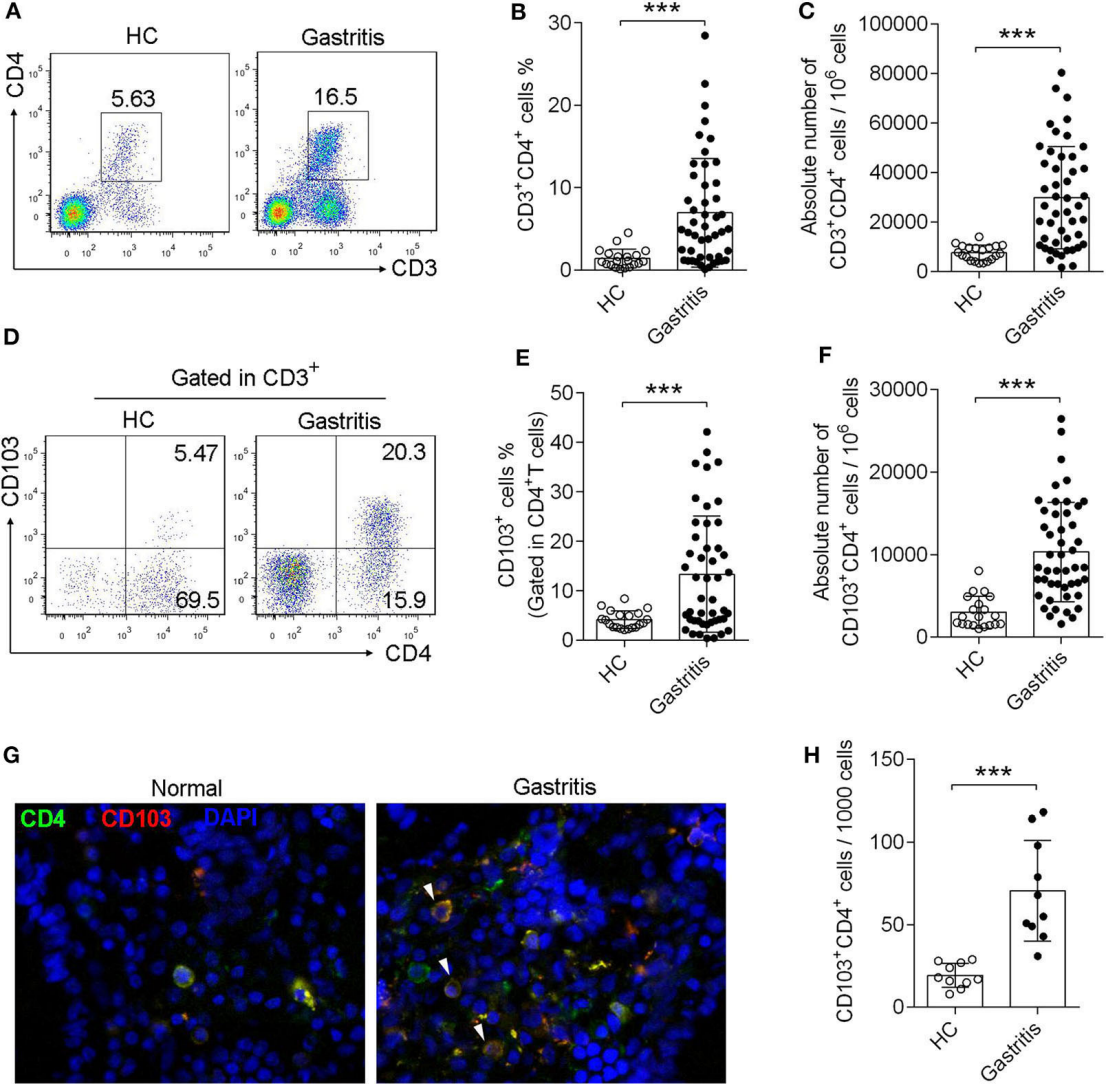
Induction of CD103^+^CD4^+^T cells in the gastric mucosa of *H. pylori*-positive patients. **(A–C)** The frequency **(A,B)** and absolute number **(C)** of CD3^+^ CD4^+^ T cells of gastric mucosa from *H. pylori*-infected patients (*n* = 47) and healthy control (*n* = 20) was analyzed by flow cytometry. **(D–F)** The proportion **(D,E)** and absolute number **(F)** of CD103^+^ T cells was gated in CD3^+^ CD4^+^ subpopulation. The data are shown as the mean ± SD from independent tissues obtained from *H. pylori*-positive patients (*n* = 47) and healthy control (*n* = 20). Unpaired Student's *t*-test was performed to compare the gastritis and normal groups, and significant differences are depicted as ***P < 0.001. **(G,H)** Mucosa tissue was double stained with anti-CD4 (Green) and anti-CD103 (Red) Abs, and then observed by fluorescent microscopy. Three independent experiments were performed with comparable results. One representative experiment is shown.

Sixty seven subjects were enrolled in this study, including 20 healthy controls with *H. pylori*-negative and 47 gastritis patients with *H. pylori*-positive, undergoing endoscopy to clarify the origin of symptoms (Table 1). Forty seven subject were diagnosed with *H. pylori*-positive gastritis with pathological changes of mucosa, whereas 20 subjects were diagnosed with functional bowel disease (gastrointestinal disease without pathological changes, used as healthy control) with *H. pylori*-negative and normal mucosa (Table 1). First, we found that the CD4^+^T cell frequency and absolute numbers were increased in the gastric mucosa from *H. pylori*-infected patients compared with those of the normal control (Figures 1A–C). We next analyzed CD103 expression in gastric CD4^+^T cells by flow cytometry. Flow cytometry data showed that CD103 was highly expressed in gastric CD4^+^ T cells from *H. pylori*-positive gastritis compared to with that in the normal control (Figure 1D). CD4^+^T cells in the gastric mucosa showed a high frequency of CD103^+^ cells (21.43% ± 1.73%), while comprising only 4.16% (4.16% ± 0.39%) of total CD4^+^T cells in normal mucosa (Figure 1E). In addition, absolute number of CD103^+^CD4^+^T cells was increased in the gastric mucosa (Figure 1F). Immunofluorescence staining showed increased number of CD103 expressed-CD4^+^T cells in gastric tissue compared with that in the normal control (Figures 1G,H). Together, these results demonstrated that CD103^+^CD4^+^ T cells were increased in the gastric mucosa of patients with *H. pylori*-positive gastritis.

**Table 1:** Characteristics of healthy donors and gastritis patients.

	**Healthy**	**Gastritis**	***P-*value**
Sample size (no.)	20	47	–
Age (years) (mean ± SD)	42.35 ± 16.79	38.72 ± 14.51	0.375
Sex (M/F)	12/8	25/22	0.789
**Indication for endoscopy (%)**			
Recurrent abdominal pain	12 (60)	18 (38.3)	0.116
Burning abdominal discomfort	2 (10)	2 (4.3)	0.577
Acid reflux symptoms	1 (5)	9 (8.5)	0.260
Dyspepsia	0 (0)	5 (10.6)	0.312
Peptic ulcer	0 (0)	6 (12.8)	0.168
Epigastric pain	1 (5)	8 (19.1)	0.256
**Endoscopic finding (%)**			
Normal	20 (100)	0 (100)	<0.0001
Gastritis	0 (0)	47 (100)	<0.0001
^13^C-urea breath test positive (DOB>5) (%)	0 (0)	47 (100)	<0.0001
*H. pylori* infection (%)	0 (0)	47 (100)	<0.0001

*F, Female; M, male; DOB, Delta Over Baseline. The level of significance was evaluated by unpaired student t-test or Chi-square test. P < 0.05 was considered statistically significant*.

### Memory-Like Phenotype and High Chemokine Receptors Expression Was Observed in Gastric CD103^+^CD4^+^T Cells of *H. pylori*-Positive Patients

**Figure 2:**
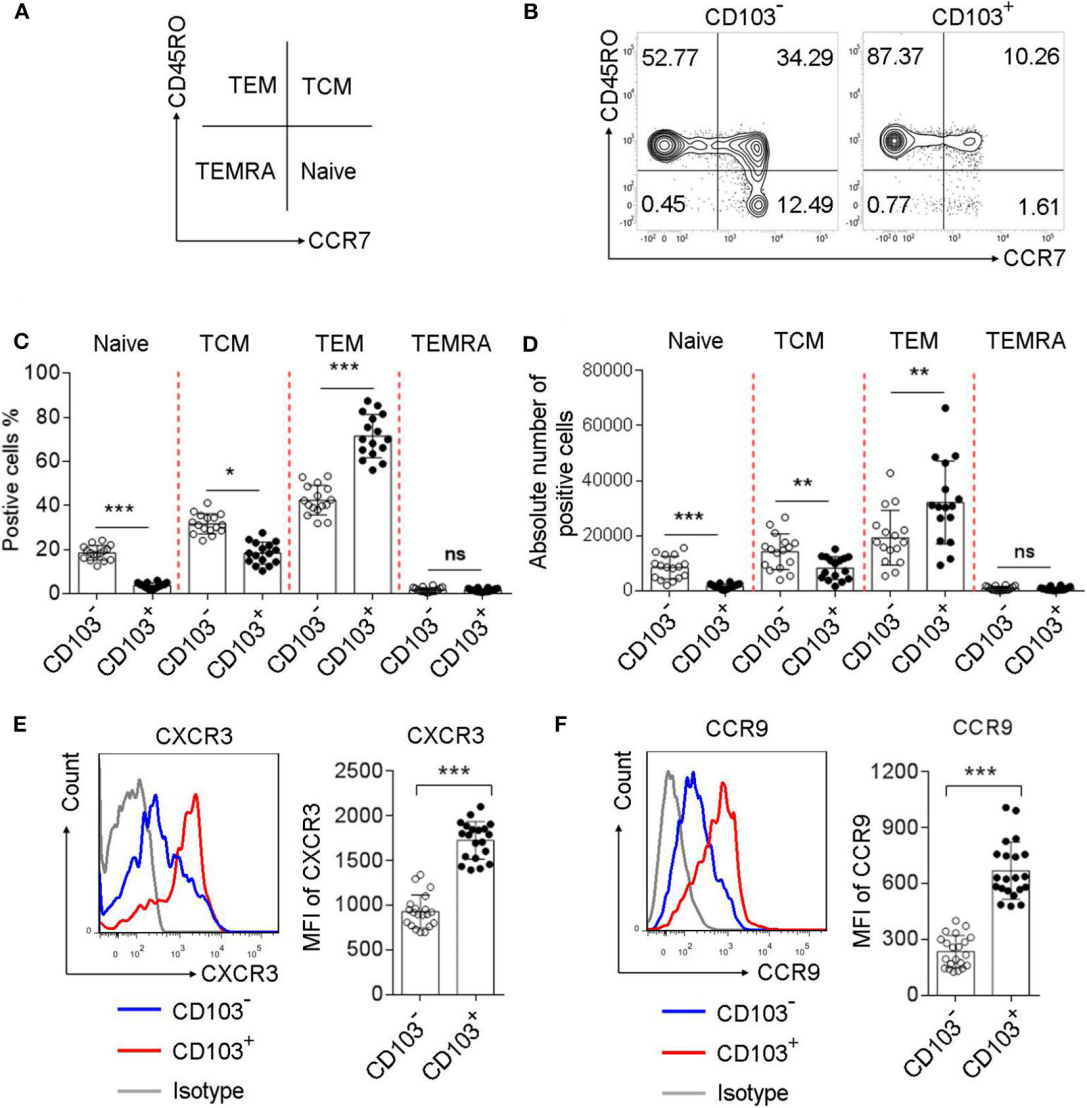
Increased memory and chemokine status in gastric CD103^+^CD4^+^T cells of *H. pylori*-positive patients. **(A–D)** Flow cytometry analysis with CD45RO/CCR7 staining to define the T cell subsets in CD103^+^ vs. CD103^−^ CD4^+^ T cells from *H. pylori*-positive patients (*n* = 16). The percentages **(C)** and absolute number **(D)** of naive T cells, T_EM_, T_CM_ and terminally differentiated effector cells T_EMRA_ were compared in CD103^+^ vs. CD103^−^ CD4^+^T cells. **(E,F)** The expression levels of chemokine receptors, including CXCR3 **(E)** and CCR9 **(F)** were determined by flow cytometry in CD103^+^ or CD103^−^CD4^+^T cells from *H. pylori*-positive patients (*n* = 20). The mean fluorescence intensity (MFI) of CXCR3 and CCR9 for each indicated marker in CD103^+^ vs. CD103^−^ CD4^+^T cells was compared. The data are shown as the mean ± SD from independent tissues obtained from *H. pylori*-positive patients. Unpaired Student's *t*-test was performed to compare the CD103^+^ and CD103^−^ groups, and significant differences are depicted as *P < 0.05, **P < 0.01, and ***P < 0.001. ns, not significant (*P* > 0.05).

To investigate the phenotype of gastric CD4^+^T cells expressing CD103, we analyzed the percentage of naive T cells (CD45RO^−^CCR7^+^), central memory T cells (T_CM_, CD45RO^+^CCR7^+^), effector memory T cells (T_EM_, CD45RO^+^CCR7^−^), and terminally differentiated effector T cells (T_EMRA_, CD45RO^−^CCR7^−^), based on the memory marker CD45RO and lymph node homing receptor CCR7(Figure 2A). The frequency of T_EM_ was increased, while the percentages of T_CM_ and naïve T cells were decreased, in CD103^+^ CD4^+^ vs. CD103^−^ CD4^+^ T cells from the gastric mucosa of *H. pylori*-positive patients (Figures 2B,C). In addition, the absolute number of T_EM_ was increased, while the absolute number of T_CM_ and naïve T cells were decreased, in CD103^+^ CD4^+^ vs. CD103^−^ CD4^+^ T cells (Figure 2D). We next explored whether gastric CD103^+^CD4^+^T cells exhibited distinct chemokine phenotypes. The results showed upregulation of the surface molecules CXCR3 and CCR9 on CD103^+^CD4^+^T cells compared with those on CD103^−^CD4^+^T cells (Figures 2E,F). The mean fluorescence intensities (MFI) of CXCR3 and CCR9 were also significantly upregulated in CD103^+^CD4^+^T cells compared with those of CXCR3 and CCR9 on CD103^−^CD4^+^T cells (Figures 2E,F). Together, these results indicated CD103^+^CD4^+^T cells as a subset of effector memory T cells in the gastric mucosa of *H. pylori*-positive patients.

### CD103 Promoted the Proliferation of Gastric CD4^+^T Cells Induced by *H. pylori* Antigen-Primed DCs

**Figure 3:**
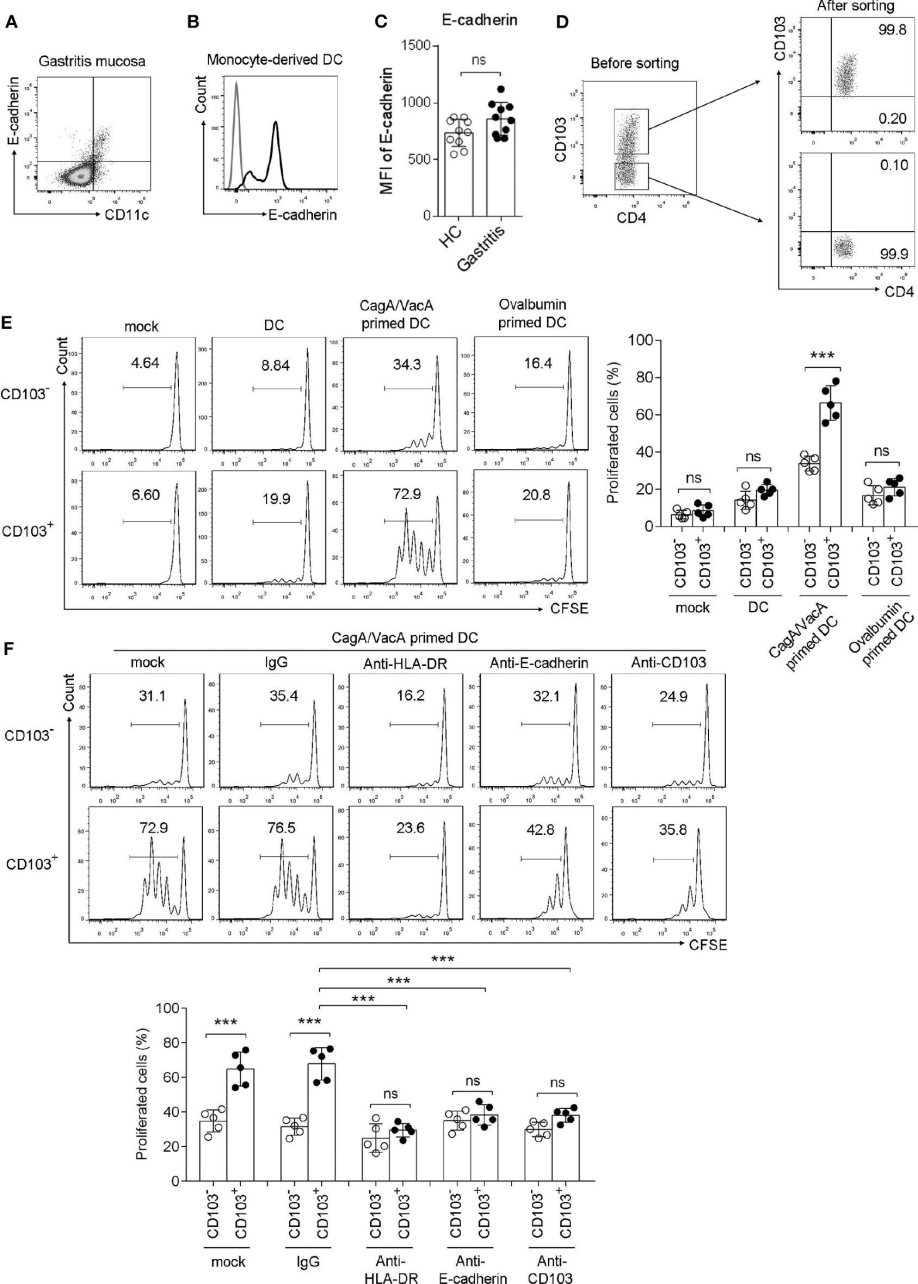
CD103 promoted the proliferation of gastric CD4^+^T cells induced by *H. pylori* antigen-primed DCs. **(A,B)** The expression level of E-cadherin was determined by flow cytometry in gastric CD11c^+^ cells **(A)** from *H. pylori*-positive patients and monocyte-derived DCs **(B)**. **(C)** The MFI of E-cadherin was analyzed in gastric CD11c^+^ cells from *H. pylori*-positive patients (*n* = 10) and healthy control (*n* = 10). **(D)** The purify of sorted CD103^+^ and CD103^−^ CD4^+^T cell was analyzed by using flow cytometry. **(E)** CagA and VacA-primed autologous DCs were cocultured with CFSE-labeled mucosal T cells for 5 days at an APC/responder cell ratio of 1:10. **(F)** Autologous DCs were incubated with CagA and VacA then cocultured with mucosal T cells in the presence of anti-HLA-DR, anti-E-cadherin, anti-CD103, or isotype control antibodies. Proliferation gated CD103^+^ or CD103^−^CD4^+^T cells were determined by flow cytometry. Three independent experiments were performed with comparable results. One representative experiment is shown. The significant differences are depicted as ***P < 0.001. ns, not significant (*P* > 0.05).

To determine whether gastric CD103^+^CD4^+^ T cells respond to the *H. pylori* antigen, we performed a coculture assay. First, we determined the CD103 ligand E-cadherin expression in DCs, which are the major antigen-presenting cells in T cell activation. The data showed that E-cadherin was highly expressed in gastric DCs from *H. pylori*-positive patients and monocyte-derived DCs *in vitro* (Figures 3A,B). However, *H. pylori* did not upregulated E-cadherin expression in gastric DCs (Figure 3C). Gastric CD103^+^ and CD103^−^CD4^+^ T cell was sorted by using flow cytometry (Figure 3D). Next, monocyte-derived DCs were treated with *H. pylori* virulence factor VacA and CagA purified protein for 24 h, then cocultured with autologous CFSE-labeled CD103^+^ vs. CD103^−^CD4^+^T cells for 3 days. The percentage of proliferated T cells was assessed in CD103^+^CD4^+^ and CD103^−^CD4^+^T cells. Sorted CD103^+^CD4^+^T cells cocultured with VacA/CagA-primed DCs exhibited increased proliferation, while these cells had few proliferation upon untreated DC and ovalbumin-primed DC (Figure 3E). We also found that CD103^−^CD4^+^T cells activated by VacA/CagA-primed DCs had low proliferation compared with that of CD103^+^CD4^+^T cells (Figure 3F). Neutralization of the antigen-presenting molecule HLA-DR completely blocked CD103^+^CD4^+^T cell proliferation (Figure 3F). Furthermore, blocking E-cadherin-CD103 interaction with anti-E-cadherin or anti-CD103 antibody reduced CD103^+^CD4^+^ T cell proliferation in the coculture system (Figure 3F). Thus, we found that CD103 promoted gastric CD4^+^ T cell response upon stimulation with VacA/CagA-primed DCs.

### CD103 Enhanced IFN-γ, TNF, and IL-17a Production by Gastric CD4^+^T Cells From *H. pylori*-Positive Patients

**Figure 4:**
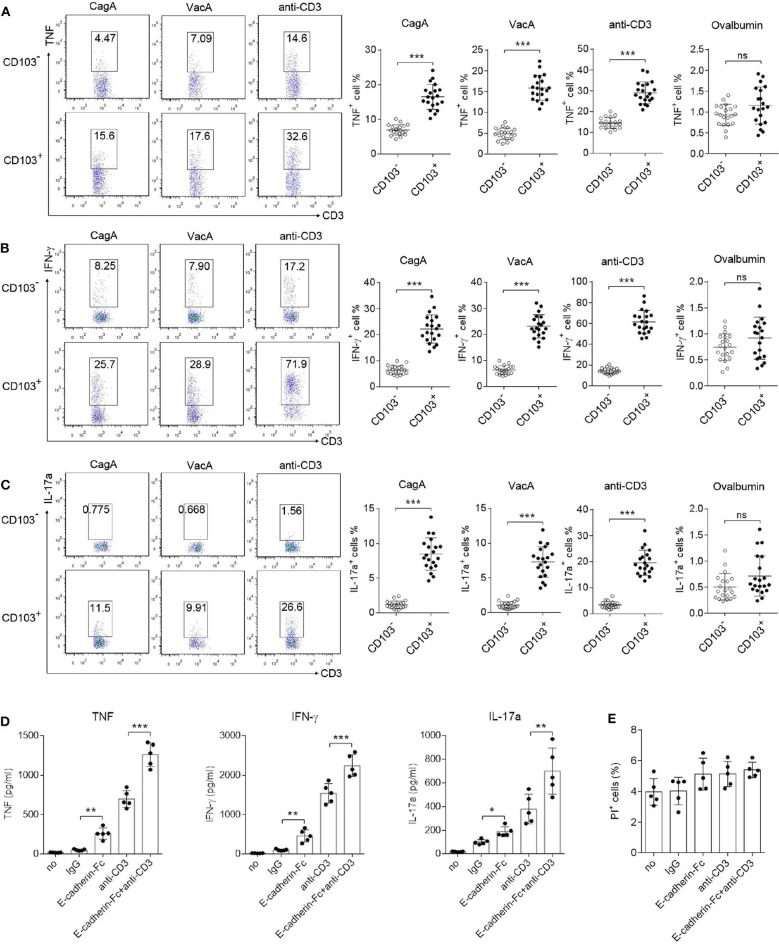
CD103 enhanced IFN-γ, TNF, and IL-17a production by gastric CD4^+^T cells from *H. pylori* positive patients. **(A–C)** Mucosal lymphocytes from *H. pylori*-positive patients were stimulated with anti-CD3 (1 μg/ml), or VagA (10 μg/ml) or CagA (10 μg/ml), or ovalbumin (10 μg/ml) for 12 h. The percentages of TNF-**(A)**, IFN-γ-**(B)**, and IL-17a-**(C)** producing T cells in CD103^+^ or CD103^−^ CD4^+^T cells were detected by flow cytometry. The data are shown as the mean ± SD from independent tissues obtained from *H. pylori*-positive patients (*n* = 20). **(D,E)** Gastric CD4^+^T cells from *H. pylori* positive patients (*n* = 5) were stimulated with E-cadherin-Fc fusion protein (10 μg/ml), anti-CD3 (1 μg/ml), or control IgG for 24 h. **(D)** Concentrations of IFN-γ, TNF, and IL-17a in culture supernatant were determined by ELISA. **(E)** Propidium iodide (PI) positive cells were determined by flow cytometry. Unpaired Student's *t*-test was performed to compare the CD103^+^ and CD103^−^ groups, and significant differences are depicted as *P < 0.05, **P < 0.01, ***P < 0.001.

Because Th1/Th17 cells mediate pro-inflammatory responses against *H. pylori* infection (Velin et al., 2009; Shi et al., 2010), we next evaluated IFN-γ, TNF and IL-17a production by CD103^+^CD4^+^T cells. Mononuclear cells isolated from the gastric mucosa were stimulated with CagA or VacA recombinant protein, or anti-CD3 antibody (Ab) for 12 h. Flow cytometry data showed that upon CagA or VacA stimulation, the production of IFN-γ, TNF and IL-17a was increased in CD103^+^ vs. CD103^−^CD4^+^ T cells (Figures 4A–C). We also found that CD103^+^CD4^+^T cells stimulated by the anti-CD3 Ab (a type of antigen-nonspecific stimulation) showed high levels of TNF, IFN-γ, and IL-17a compared with those in CD103^−^CD4^+^ T cells (Figures 4A–C). Furthermore, the effect was confirmed by antigen non-specific stimulus, gastric mononuclear cells were stimulated with ovalbumin recombinant protein. Both CD103^−^ and CD103^+^CD4^+^T cells produced few levels of TNF, IFN-γ and IL-17a upon ovalbumin treatment (Figures 4A–C). To analyze the direct role of CD103 signaling in gastric CD4^+^T cells, the E-cadherin-Fc fusion protein alone or combined with the anti-CD3 Ab were used to stimulate sorted gastric CD4^+^T cells for 24 h. ELISA data showed that E-cadherin-Fc alone induced slightly higher TNF, IFN-γ, and IL-17a levels in gastric CD4^+^T cells compared with those induced by the IgG control (Figure 4D). Furthermore, E-cadherin-Fc synergized with the anti-CD3 Ab significantly increased the TNF, IFN-γ, and IL-17a concentrations in gastric CD4^+^T cells compared with those induced by the anti-CD3 alone (Figure 4D). Meanwhile, E-cadherin-Fc did not induced cell death of gastric CD4^+^T cells (Figure 4E). Taken together, CD103 promoted pro-inflammatory cytokines produced by gastric CD4^+^T cells from *H. pylori*-positive patients.

### CD103 Interacted With TCRα/β to Amplify the TCR/CD3ζ/ZAP70 Signaling Pathway in Gastric CD4^+^T Cells Induced by TCR Stimulation

**Figure 5:**
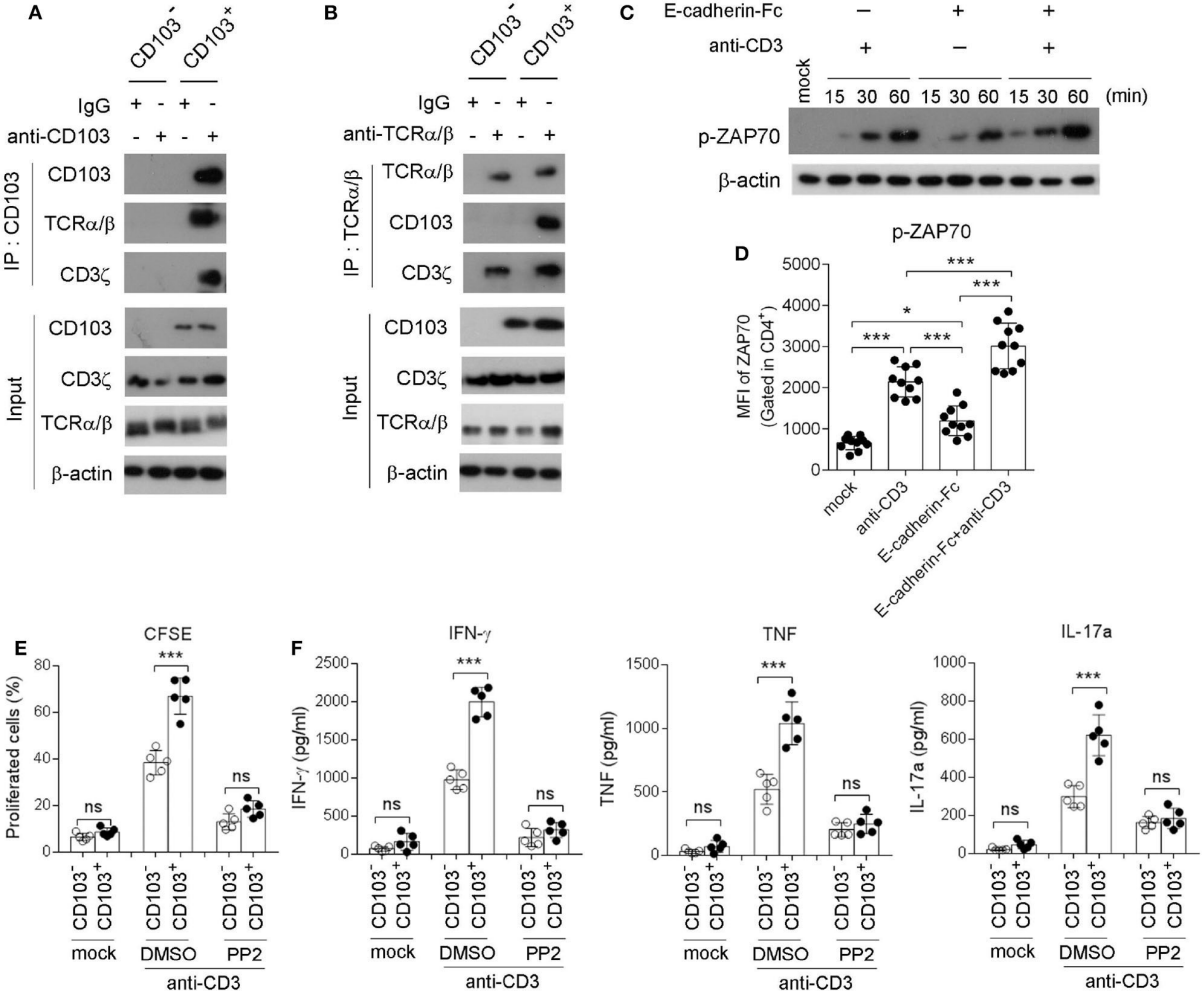
CD103 interacted with TCRα/β/CD3ζ to enhance ZAP70 signaling in gastric CD4^+^T cells. **(A,B)** CD103^+^ or CD103^−^ CD4^+^T cells were isolated from gastric tissue of *H. pylori*-positive patients. Blots of cell lysates (input), anti-CD103 **(A)** or anti-TCR **(B)** immunoprecipitates were analyzed for CD103, CD3ζ or TCR by western blot. **(C,D)** Gastric CD4^+^T cells were treated with anti-CD3 (1 μg/ml) or E-cadherin-Fc fusion protein (10 μg/ml). Phosphorylated ZAP70 (Tyr315) was analyzed by western blot **(C)** and flow cytometry **(D**, *n* = 10). **(E,F)** CD103^+^ or CD103^−^ CD4^+^ T cells (*n* = 5) sorted from gastric tissue were treated with anti-CD3 (1 μg/ml) in the presence of PP2 (1 μM) or DMSO. **(E)** Cell proliferation was determined by flow cytometry. **(F)** Concentrations of IFN-γ, TNF, and IL-17a in culture supernatant were determined by ELISA. Data are representative of three independent experiments. ns, no significant; *P < 0.05, ***P < 0.001.

Next, immunoprecipitation (IP) was applied to explore the downstream signaling of CD103 in gastric CD4^+^ T cells. Anti-CD103 IP was performed in sorted gastric CD4^+^ T cells, and then immunoblots were analyzed with specific Ab against CD103, TCRα/β, and CD3ζ. Our results indicated that CD103 interacted with TCRα/β and CD3ζ in gastric CD4^+^ T cells (Figure 5A). To confirm the interaction between CD103 and TCRα/β anti-TCRα/β IP was performed in sorted gastric CD4^+^ T cells, and CD103 and TCRα/β were analyzed by immunoblots. The data revealed that TCRα/β interacted with CD103 and CD3ζ in gastric CD4^+^ T cells (Figure 5B). Next, western-blot and flow cytometry assays were applied to assess the phosphorylation of kinase ZAP70 (Tyr319) in gastric CD4^+^ T cells treated with the E-cadherin-Fc fusion protein or the anti-CD3 Ab. The CD103 signal activated by the E-cadherin-Fc fusion protein induced the phosphorylation of ZAP70 (Tyr319) (Figure 5C). Flow cytometry also showed that the phosphorylation of ZAP70 (Tyr319) was increased in gastric CD4^+^T cells after stimulation with anti-CD3 Ab and the E-cadherin-Fc fusion protein (Figure 5D). The E-cadherin-Fc fusion protein combined with anti-CD3 Ab enhanced the phosphorylation of ZAP70 (Tyr319) compared with those induced by anti-CD3 Ab alone (Figures 5C,D). Together, these results indicated that CD103 interacted with TCRα/β and CD3ζ to amplify the ZAP70 signal pathway in gastric CD4^+^T cells.

To investigate whether the TCR/CD3ζ/ZAP70 pathway was essential for the CD103-mediated gastric CD4^+^T cell response, we determined the proliferation and cytokine production by gastric CD103^+^CD4^+^T cell pretreatment with the ZAP70 inhibitor PP2 (Figures 5E,F). Flow cytometry showed that the proliferation of gastric CD103^+^CD4^+^T cells was blocked by pretreatment with PP2 (Figure 5E). Consistently, we found that CD103^+^CD4^+^ T cells produced higher IFN-γ, TNF and IL-17a levels compared with those induced by CD103^−^CD4^+^ T cells stimulated with the anti-CD3 Ab, whereas this effect was compromised by pretreatment with PP2 (Figure 5F). The results indicated that CD103 enhanced gastric CD4^+^T cell response dependent on ZAP70 signaling pathway.

### CD103 Upregulated T-bet and Blimp1 Expression in Gastric CD4^+^T Cells Dependent on the CD3ζ/ZAP70 Pathway

**Figure 6:**
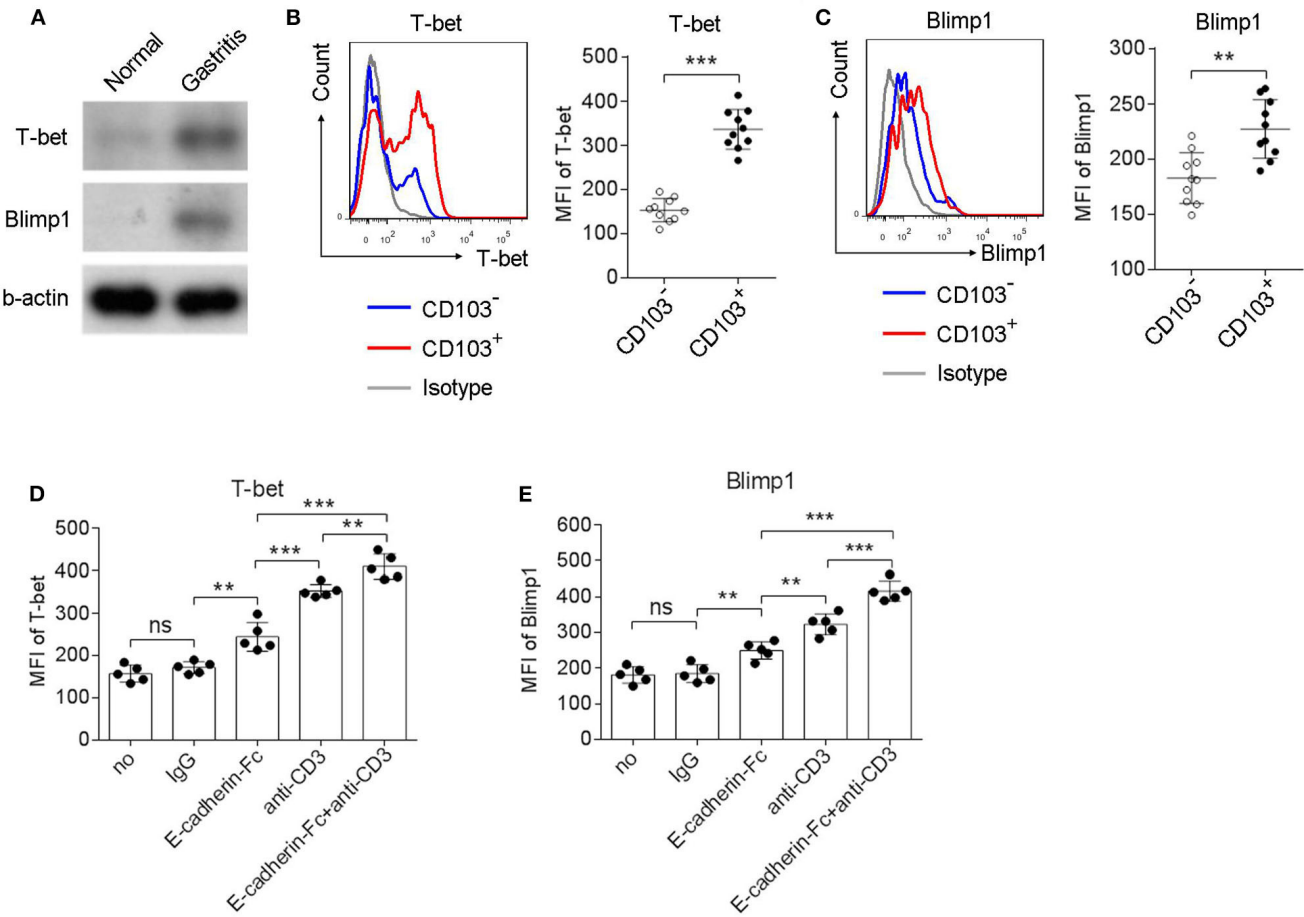
CD103 upregulated Blimp1 and T-bet expression in gastric CD4^+^T cells dependent on the CD3ζ/ZAP70 pathway. **(A)** Normal and gastric tissue was collected from healthy control and *H. pylori*-positive patient, respectively, and then were analyzed for T-bet and Blimp1 by western blot. **(B,C)** Gastric lymphocytes (*n* = 10) were stimulated with anti-CD3/CD28 (1 μg/ml) for 12 h. The expression levels of T-bet **(B)** and Blimp1 **(C)** in CD103^+^ or CD103^−^ CD4^+^ T cells were analyzed by flow cytometry. **(D,E)** Gastric CD4^+^T cells (*n* = 5) were treated with anti-CD3 (1 μg/ml) and E-cadherin-Fc fusion protein (10 μg/ml) for 24 h. The expression levels of T-bet **(D)** and Blimp1 **(E)** in CD4^+^ T cells were analyzed by flow cytometry. The data are shown as the mean fluorescence intensity (MFI) of indicated molecules. Unpaired Student's *t*-test was performed to compare the CD103^+^ and CD103^−^ groups, and One-way ANOVA test was performed to compare each groups in **(D,E)**. Significant differences are depicted as **P < 0.01, ***P < 0.001.

Homolog of B lymphocyte-induced maturation protein (Blimp1) and the T-box transcription factor T-bet has been reported to be expressed in tissue resident memory T cells (Topham and Reilly, 2018). Therefore, we next explored whether CD103^+^CD4^+^T cells expressed Blimp1 and T-bet. Upregulation of Blimp1 and T-bet was observed in the gastric mucosa compared to those in the normal control of *H. pylori*-positive patients (Figure 6A). In addition, the Blimp1 and T-bet levels were also higher in CD103^+^CD4^+^T cells compared with those in CD103^−^CD4^+^T cells (Figures 6B,C). The MFI of Blimp1 (Figure 6B) and T-bet (Figure 6C) was significantly upregulated in CD103^+^CD4^+^ T cells compared with those of Blimp1 and T-bet in CD103^−^CD4^+^T cells. The expression of T-bet and Blimp1 was increased in gastric CD4^+^T cells after stimulation with the anti-CD3 Ab and the E-cadherin-Fc fusion protein (Figures 6D,E). Furthermore, E-cadherin-Fc synergized with anti-CD3 Ab significantly increased the expression of T-bet and Blimp1 in gastric CD4^+^T cells compared with those induced by anti-CD3 alone (Figures 6D,E). These data suggested that CD103 increased Blimp1 and T-bet expression in gastric CD4^+^ T cells dependent on the CD3ζ/ZAP70 pathway.

## Discussion

CD103 is an adhesion molecule expressed by immune cells that presumably contributes to their tissue-specific localization (Corgnac et al., 2018). In this study, we demonstrated that CD103 provided a intrinsic costimulatory signal to gastric CD4^+^T cells from *H. pylori-*positive patients, and enhanced IFN-γ, TNF, and IL-17a production upon *H. pylori* antigen stimulation. This study explored a novel role of CD103 on gastricCD4^+^ T cell response upon *H. pylori* infection, which may explain the local inflammatory response of peripheral tissues during infection and injury.

As a member of the integrin family, CD103 (also known as αE integrin) broadly expressed in various immune cells. CD103 is expressed at high levels on T cells in the skin and eyes, and in the mucosa of the gut and lungs of healthy patients, as well as on dendritic cells, innate lymphoid cells and natural killer cells (Sathaliyawala et al., 2013). After migration to mucosal tissue, CD103 expression on T cells is induced and maintained by TGF-β produced by epithelial and dendritic cells (Zhang and Bevan, 2013). Increasing evidence shows that CD103 is induced by infection or inflammation in both tissue resident CD4^+^ and CD8^+^ T cells (Allez et al., 2002). Previous studies have indicated that CD103 is expressed in gastric CD4^+^ and CD8^+^ T cells, which were identified as tissue resident memory T cells (Booth et al., 2014). In the study, we found that CD103 was upregulated in gastric CD4^+^ T cells from *H. pylori* positive patients.

CD103 exerts its function through binding to E-cadherin on intestinal epithelial cells (IECs) and mediating cell adhesion and retention of T cells within the mucosa (Schon et al., 1999). The migration of immune cells from the peripheral blood to mucosa tissues is driven by the expression of tissue-specific homing receptors, such as CXCR3 and CCR9 (Iwata et al., 2004; Annunziato et al., 2006; Cassani et al., 2011). CXCR3 selectively binds to three chemokines, CXCL9, CXCL10, and CXCL11, which are induced by the IFN-γ signal (Jinquan et al., 2000). CCR9 was differentially expressed on T lymphocytes of small intestine and colon, and the specific ligand is CCL25 (Wurbel et al., 2001). In our study, we found that CXCR3 and CCR9 were significantly upregulated in CD103^+^CD4^+^T cells compared with those in CD103^−^CD4^+^T cells in the gastric mucosa of *H. pylori*-positive patients. Moreover, we found that CD103 expression on naive CD4^+^ T cells was substantially lower than that in effector and memory T cell subsets (T_CM_, T_EM_). These results indicated that CD103^+^CD4^+^T cells may be a effector memory T cell subset with mucosal chemotaxis.

Except for the adhesion of integrin, CD103 may act as a regulatory molecule in T cells. The interaction of CD103 with E-cadherin on tumor cells optimizes cytokine release in CD8^+^ T cells, since siRNA targeting E-cadherin partially inhibits IFN-γ and granzyme B production (Franciszkiewicz et al., 2013). Consistently, the cytotoxicity of CD103^+^ tumor-infiltrating lymphocyte (TIL) toward autologous E-cadherin^+^ tumor cells is inhibited anti-CD103 blocking antibody (Le Floc'h et al., 2007). However, several studies have indicated that CD103 may define a subset of T cells with regulatory activity (Braun et al., 2015; Zhong et al., 2018). However, CD4^+^T_reg_ expressing CD103 are crucial for the regulation of murine contact hypersensitivity, as CD103-deficient T_reg_ are unable to suppress allergic skin inflammation (Braun et al., 2015). Thus, the intrinsic role of CD103 in T cells was controversial, which may be needed for further study.

*H. pylori* infection leads to an enhanced expression of antigen-presenting molecules of APCs in the lamina propria (Krauss-Etschmann et al., 2005), which may represent an effort of the immune system to optimize local immune responses against *H. pylori*. In our study, we cocultured gastric CD103^+^CD4^+^T cells with *H. pylori-*specific antigen (CagA and VacA)-primed DCs, a classical APCs that express high levels of E-cadherin. CD103^+^CD4^+^T cells cocultured with VacA/CagA-primed DCs showed enhanced proliferation in HLA-DR-dependent manner. Gastric CD4^+^T cells secreted Th1 cytokines (IFN-γ and TNF) and Th17 cytokines (IL-17a and IL-23) when stimulated with PMA/Ionomycin (Bamford et al., 1998; Caruso et al., 2008). Recently, Booth et al. (2014) reported that gastric CD4^+^T_RM_ (~40% of CD103^+^) cells obtained from biopsies were responsive to staphylococcal enterotoxin B and anti-CD3/CD28 bead stimulations by secreting Th1 and Th17 cytokines including IFN-γ, TNF, and IL-17a. Furthermore, upon CagA or VacA stimulation, the production of IFN-γ, TNF, and IL-17a was increased in CD103^+^ vs. CD103^−^CD4^+^ T cells. Using the E-cadherin-Fc fusion protein or anti-E-cadherin blocking Ab, we found that CD103 signal promoted proliferation and IFN-γ, TNF, and IL-17a production by gastric CD4^+^T cells. Together, we suggested that CD103 induced a predominant pro-inflammatory response of gastric CD4^+^ T cell during *H. pylori* infection.

CD103-E-cadherin tight adhesion promotes the phosphorylation of proline-rich tyrosine kinase-2 (Pyk2), and subsequent binding of phosphorylated-paxillin to the CD103 subunit tail (Gauthier et al., 2017). In addition, the adhesive interaction of E-cadherin with CD103 on CD8^+^TIL triggers the phosphorylation of extracellular signal-regulated kinases 1 and 2 (ERK1/2) and phospholipase Cγ1 (PLCγ1), providing intracellular signals that promote CD8^+^CTL effector functions (Le Floc'h et al., 2011).

In the study, we found that CD103 interacted with TCRα/β in gastric CD4^+^ T cells, which indicated that CD103 may be a component of immunological synapse. It is well-known that Ag-specific signals via TCR induces the phosphorylation of immunoreceptor tyrosine-based activation motif (ITAM) on CD3 chains, particularly the CD3ζ, and then recruits the kinase Zap70 to the phosphorylated ITAMs (van Oers et al., 2000). Activated ZAP70 in turn phosphorylates and activates various downstream signal transduction molecules, leading to T cell activation (Klammt et al., 2015). We found that stimulation with the E-cadherin-Fc fusion protein increased the phosphorylation of ZAP70 in gastric CD4^+^T cells. Furthermore, proliferation as well as IFN-γ, TNF, and IL-17a production were increased in CD103^+^ vs. CD103^−^ CD4^+^ T cells and these effects were alleviated by the ZAP70 inhibitor PP2. We suggest that intrinsic CD103 functioned as a costimulatory molecule to promoted the TCR-mediated CD3ζ/ZAP70 signaling pathway.

The different transcription factor profile of CD103^+^CD4^+^T_RM_ cell subsets contributed to the functional heterogeneity. Here, we speculated that T-bet and Blimp1 were key transcription factor for gastric CD103^+^CD4^+^T cell. T-bet is a key regulator of effector memory T cells, but T-bet is downregulated in CD103^+^CD8^+^T_RM_ in the skin, gut, lung, and brain (Wakim et al., 2012). However, a moderate level of T-bet expression is essential for CD122 expression and IL-15 responsiveness in CD103^+^CD8^+^T_RM_ (Mackay et al., 2015). Blimp1 is a transcription factor that is broadly expressed in the effector stages of multiple hematopoietic lineages including plasma cells, T cells and NK cells (Cretney et al., 2011). Importantly, Blimp1 regulates the genes important for residence of T_RM_, and plays a critical role in regulating effector function (Mackay et al., 2016). The loss of both T-bet and Blimp-1 leads to abrogated cytotoxic function and ectopic IL-17 production in CD8^+^T cells (Xin et al., 2016), which indicated that effector CD8^+^T cell differentiation was governed by the availability of Blimp-1 and T-bet. In the study, we found that gastric CD103^+^CD4^+^T cell expressed higher levels of T-bet and Blimp1, which was enhanced by E-cadherin-Fc. Thus, we supposed that CD103 regulated T-bet and Blimp1in gastric CD4^+^T cells upon *H. pylori* infection.

In summary, we demonstrated that CD103 was induced in gastric CD4^+^T cells of *H. pylori*-positive patients. The CD103^+^ CD4^+^T cell subset exhibited high expression and activation of memory-related molecules, as well as the chemokine receptor CXCR3 and CCR9. Furthermore, IFN-γ, TNF, and IL-17α production as well as proliferation were significantly increased in CD103^+^CD4^+^ T cells compared with those in CD103^−^CD4^+^T cells in the coculture with CagA and VacA-primed DCs. CD103 interacted with TCRα/β and enhanced CD3ζ/ZAP70 signaling, which was essential for proliferation and pro-inflammatory cytokine production by gastric CD4^+^T cells. Moreover, CD103 promoted T-bet and Blimp1 expression in gastric CD4^+^T cells. Our results explore the function of CD103 in gastric CD4^+^T cell of *H. pylori*-positive patients, which may provide a therapeutic target for the treatment of gastritis.

## Materials and Methods

### Ethics Statement

The study was approved by the Ethics Committee of the First Affiliated Hospital of Jinan University and Guangzhou Women and Children's Medical Center, Guangzhou Medical University (approval number 2017021709). Biopsy specimens from the *H. pylori*-positive patients were collected from Guangzhou Women and Children's Medical Center (Guangzhou, China) and the First Affiliated Hospital of Jinan University (Guangzhou, China). Informed written consent was obtained from participants prior to commencement of the study.

### Subjects

The study included 20 healthy donor and 47 gastritis patients residing in Guangzhou (Table 1) undergoing evaluation for chronic symptoms suggestive of peptic disease, including dyspepsia and recurrent abdominal discomfort and pain. Exclusion criteria included a history of acute onset of symptoms, acute or chronic vomiting, or the use of antibiotic, antacid, H2 blockers, proton-pump inhibitors, bismuth-containing compounds, or non-steroidal anti-inflammatory drugs within the preceding 4 weeks. Biopsy samples were obtained from the patients with endoscopy indications for histopathological examinations (Table 1). The samples were stained by Giemsa dye to observe the bacteria under a light microscopy. Both 47 patients was *H. pylori*-positive, while 20 healthy controls were *H. pylori*-negative. Patients with the diameter >5 mm and a certain depth damaged stomach mucosal tissue were defined as peptic ulcer (in the presence of exudates), while gastritis was diagnosed in patients with damaged mucosal tissue <5 mm in diameter.

### Isolation of Mucosal Mononuclear Cells

Biopsy specimens were obtained from the antral and oxyntic gastric mucosa of all patients. The biopsy tissue isolation procedure used in this study was previously described (Booth et al., 2014). The enzymatic digestion solution consisted of 1 ml of RPMI 1640 (Gibco) containing, 10 μl of Collagenase D (100 μg/ml; Sigma-Aldrich, St. Louis, MO, USA) and 1 μl of DNase I (10 μg/ml; Thermo Fisher Scientific, Waltham, MA, USA). The biopsies were digested for 45 min at 37°C with shaking. After the digestion, the cells were collected in a 15 ml tube through a 70 μm cell strainer and centrifuged at 1,500 rpm. The cells were then washed and re-suspended in basic RPMI 1640. A total of 1 × 10^6^ cells were stained immediately for immunophenotyping by flow cytometry (see below).

### Flow Cytometric Analysis

The cell staining procedure used in this study was described previously (Wu et al., 2018a). For intracellular cytokine staining, the cells were restimulated for 12 h with 10 g/ml CagA (Linc-Bio Science, Shanghai, China), 10 μg/ml VacA (Linc-Bio Science, Shanghai, China), anti-CD3 mAb (Clone UCHT1, BD), anti-CD28 mAb (Clone CD28.2, BD) and 3 μg/ml brefeldin A (eBioscience, CA, USA). Intracellular cytokines were stained using the intracellular fixation/permeabilization buffer set (eBioscience, CA, USA). Flow cytometric analysis was performed on FACS Canto II (BD, NJ, USA), and the data were analyzed using FlowJo software (Tree Star). The following anti-human antibodies were purchased from eBioscience, BD Biosciences or Biolegend (CA, USA): CD3 (Clone UCHT1, BD), CD4 (Clone L200, BD), CD8 (Clone RPA-T8, BD), CD45RO (Clone UCHL1, BD), CD69 (Clone FN50, Biolegend), CD103 (Clone Ber-ACT8, Biolegend), CCR7 (Clone 150503, BD), CCR9 (Clone L053E8, Biolegend), CXCR3 (Clone 1C6/CXCR, BD), E-Cadherin (Clone DECMA-1, Biolegend), TNF (Clone MAb11, eBioscience), IFN-γ (Clone 4S.B3, eBioscience), IL-17a (Clone BL168, Biolegend), T-bet (Clone O4-46, BD), and Blimp1 (Clone 6D3, BD), ZAP70 Phospho (Tyr319) (Clone 1503310, Biolegend).

### Immunofluorescence Staining and Confocal Microscopy

Paraffin-embedded samples were cut into 5 μm sections, and processed for immunohistochemistry as previously described (Wu et al., 2018b). Briefly, tissue sections were fixed with 4% paraformaldehyde, followed by membrane permeabilization using 0.2% Triton-X-100. Then, the coverslips were incubated in 5% BSA, and were sequentially incubated with primary CD4 (Clone T4, Biolegend) or CD103 (Ber-ACT8, Biolegend) antibody and secondary Alexa Fluor® 488 secondary Ab (Thermo, Product # A-11034) or Alexa Fluor® 594 secondary Ab (Thermo, Product # A-11005) before mounting. Finally, the coverslips were observed under a ZEISS IMAGER A1 fluorescence microscope (CARL ZEISS) to capture fluorescence images.

### Western Blot

Western blot analysis was performed as described previously (Wu et al., 2018b). Cells were rinsed three times with ice-cold phosphate buffered saline (PBS, pH 7.4) and treated with lysis buffer containing 1% (v/v) protease inhibitor cocktail, 1 mM phenylmethylsulphonyl fluoride, and 1 mM DTT. Cell lysates with equivalent protein amounts (20 μg) were loaded, separated by SDS-PAGE, and then transferred to polyvinylidene difluoride (PVDF) membrane. The membranes were blocked in PBS-Tween20 (pH 7.4, 0.5% Tween20) with 5% bovine serum albumin (BSA) and then incubated overnight with the primary antibodies at 4°C. Then, the membranes were incubated with appropriate horseradish peroxidase-conjugated secondary antibodies at room temperature (RT) for 1 h and visualized with an ECL kit (KeyGEN, Nanjing, China) according to the manufacturer's instructions. The following anti-human antibodies were purchased from CST or abcam: Phospho-ZAP70 (Tyr319) (Clone 65E4, CST), ZAP70 (Tyr319) (Clone D1C10E, CST), TCRα/β (Clone R73, abcam), T-bet (Clone D6N8B, CST), Blimp1 (Clone C14A4, CST), CD103 (Clone Ber-ACT8, abcam), CD3ζ (Clone BL-336-1B2, abcam).

### Cells Sorting

The sorting of CD14^+^ monocyte and CD4^+^ T cells was performed as described previously (Wu et al., 2018a). Human PBMCs were used to isolate CD14^+^ cells by positive selection using the magnetic cell sorting system from BD Biosciences. Mucosal resident CD4^+^ T cell subsets were purified by positive selection using anti-human CD4 magnetic particles (BD). Mucosal resident CD103^+^ and CD103^−^ CD4^+^ T cells were sorted with a phenotype as CD3^+^CD4^+^CD103^+^ or CD3^+^CD4^+^CD103^−^ in FACSAria (BD Biosciences).

### Dendritic Cells Generation

CD14^+^ cell subsets were purified by positive selection using anti-human CD14 magnetic particles (BD). DCs were obtained from CD14^+^ monocytes after culture with granulocyte-macrophage colony-stimulating factor (GM-CSF) and IL-4 for 5 days as previously reported (Wu et al., 2018a).

### Antigen Presentation Assays

Antigen presentation assays were performed as described previously (Wu et al., 2018a). The recombinant protein CagA and VacA (10 μg/ml) were added to immature DCs for 24 h that were matured during the final 8 h with LPS. These DCs were irradiated, extensively washed and used in a coculture with CD4^+^ T cells. Sorted CD4^+^ T cells from mucosal mononuclear cells were stained with 1 μM CFSE (Invitrogen, USA) and cultured in 96-well flat-bottom plates. DCs and CD4^+^ T cells ratio were added at a ratio of 1:5 (DCs, 2 × 10^4^ cells per well; CD4^+^ T cells, 1 × 10^5^ cells per well). We calculated the percentage of divided responder T cells by gating on live CD4^+^ cells.

To study the effect of anti-CD103 and anti-E-Cadherin blocking antibody on antigen presentation assays, both CD103^+^ and CD103^−^ CD4^+^ T cells were incubated together with CagA and VacA (10 μg/ml)-primed DCs in the presence of anti-CD103 (Ber-ACT8, BD) or anti-E-Cadherin (Clone 967738, R&D Systems) blocking antibody or isotype IgG (20 μg/ml) for 5 days. Following washing and CD4 staining, T cells were tested for induction of proliferation responses.

### Statistical Analysis

Data analyses were performed in GraphPad Prism 5.0 Software (San Diego, CA, USA). Statistical significance was determined with Kruskal-Wallis or Mann-Whitney non-parametric tests or with analysis of variance (ANOVA) or Student's *t*-tests. The data are shown as the mean ± SD unless stated otherwise. A *p* < 0.05 was regarded as significant.

## Data Availability Statement

All datasets presented in this study are included in the article/supplementary material.

## Ethics Statement

The studies involving human participants were reviewed and approved by Guangzhou Women and Children's Medical Center, Guangzhou Medical University (approval number 2017021709). The patients/participants provided their written informed consent to participate in this study.

## Author Contributions

YW and SG wrote the manuscript and supervised the project. PC, SG, MW, and YW designed experiments. PC, SM, YW, JL, XN, SD, LX, and SZ performed experiments and analyzed data. CL, ZL, LG, and HW provided scientific expertise.

## Conflict of Interest

The authors declare that the research was conducted in the absence of any commercial or financial relationships that could be construed as a potential conflict of interest.
